# In Situ Synthesis of Bi_2_MoO_6_/Bi_2_SiO_5_ Heterojunction for Efficient Degrading of Persistent Pollutants

**DOI:** 10.3390/ma16103631

**Published:** 2023-05-10

**Authors:** Kaiwen Yuan, Hailong Jia, Daimei Chen, Yanmei Feng, Yu Liang, Kai Chen, Derek Hao

**Affiliations:** 1 Engineering Research Center of Ministry of Education for Geological Carbon Storage and Low Carbon Utilization of Resources, China University of Geosciences, Xueyuan Road, Haidian District, Beijing 100083, China; 2103210004@email.cugb.edu (K.Y.);; 2 School of Materials Science and Technology, Shenyang University of Chemical Technology, Shenyang 110142, China; 3 Collaborative Innovation Center of Atmospheric Environment and Equipment Technology, Jiangsu Key Laboratory of Atmospheric Environment Monitoring, Pollution Control School of Environmental Science and Engineering, Nanjing University of Information Science and Technology, Nanjing 210044, China; catqchen@163.com; 4 School of Science, STEM College, RMIT University, Melbourne, VIC 3000, Australia

**Keywords:** in situ synthesis, type II heterojunction, Bi_2_MoO_6_, Bi_2_SiO_5_, rhodamine B, tetracycline

## Abstract

Photocatalytic degradation is an environmentally friendly way to eliminate environmental pollution. Exploring a photocatalyst with high efficiency is essential. In the present study, we fabricated a Bi_2_MoO_6_/Bi_2_SiO_5_ heterojunction (BMOS) with intimate interfaces via a facile in situ synthesis method. The BMOS had much better photocatalytic performance than pure Bi_2_MoO_6_ and Bi_2_SiO_5_. The sample of BMOS-3 (3:1 molar ratio of Mo:Si) had the highest removal efficiency by the degradation of Rhodamine B (RhB) up to 75% and tetracycline (TC) up to 62% within 180 min. The increase in photocatalytic activity can be attributed to constructing high-energy electron orbitals in Bi_2_MoO_6_ to form a type II heterojunction, which increases the separation efficiencies of photogenerated carriers and transfer between the interface of Bi_2_MoO_6_ and Bi_2_SiO_5_. Moreover, electron spin resonance analysis and trapping experiments showed that the main active species were *h*^+^ and •O_2_^−^ during photodegradation. BMOS-3 maintained a stable degradation capacity of 65% (RhB) and 49% (TC) after three stability experiments. This work offers a rational strategy to build Bi-based type II heterojunctions for the efficient photodegradation of persistent pollutants.

## 1. Introduction

Persistent pollutants pose a massive potential threat to the aquatic environment due to their stable chemical nature and biological toxicity [1]. Among them, organic dyes and antibiotics are particularly polluting to the water environment, such as Rhodamine B (RhB) and tetracycline (TC). RhB can limit the penetration of sunlight, thus reducing the photosynthesis of water organisms, and it is carcinogenic, which can seriously affect human health [2]. Residues of TC in the environment cause the enrichment of resistant flora and the production of resistance genes. Unfortunately, these persistent pollutants are remarkably difficult to remove via traditional techniques [3].

Photocatalytic technology has proven to be environmentally friendly and highly efficient, and it is widely used in the degradation of persistent pollutants [4]. Currently, researchers have developed a variety of monostructural photocatalysts, such as TiO_2_ [5], MoS_2_ [6], and Bi_2_MoO_6_ [7,8]. Among them, Bi-based photocatalysts have been extensively studied for their high photoresponses, low cost, and harmfulness, as well as their controllable morphology and particular electronic band structures. One of the most well-known instances of Bi-based photocatalysts is Bi_2_MoO_6_, which consists of [MoO_6_] octahedral plates alternating with [Bi_2_O_2_] sheets [9]. It has the characteristics of non-toxicity, unique electronic structure, and suitable band gap. Its valence band comprises O 2p orbitals, and the hybridization of a large amount of Mo 4d and a small amount of Bi 6s orbitals forms the conduction band. However, a serious photogenerated charge recombination still exists, and the absorption range of visible light is less than 270 nm. In order to conquer these obstacles, the construction of Bi_2_MoO_6_ heterojunctions in combination with other suitable semiconductors has been an effective means in recent years. For example, TiO_2_/Bi_2_MoO_6_ [10,11], g-C_3_N_4_/Bi_2_MoO_6_ [12,13], and MoSe_2_/BiVO_4_ [14] have all played significant roles in solving environmental pollution. However, there have been fewer reports on the construction of heterojunctions for two structurally similar Bi-based semiconductors by using methods suitable for industrial production.

Bi_2_SiO_5_ has an excellent performance in the purification of water pollutants and the degradation of environmental pollutants. In addition, Bi_2_SiO_5_ and Bi_2_MoO_6_ are Aurivillius-structured photocatalysts, both of which are two-dimensional, layered semiconductor photocatalysts consisting of alternating [Bi_2_O_2_]^2+^ layers and anionic plates [15]. Therefore, Bi_2_SiO_5_ is appropriate for forming heterojunctions with Bi_2_MoO_6_ to address their drawbacks and enhance their photocatalytic degradation of persistent pollutants. There are various methods to synthesize Bi_2_MoO_6_ heterojunctions nowadays, such as the ion-exchange method [16], solvothermal methods [17], and the co-precipitation method [18], but the vast majority of the preparation processes utilize high-pressure hydrothermal conditions. Zhu et al. constructed compounds of Bi_2_MoO_6_/Bi_2_SiO_5_ via an anion exchange strategy between the Bi_2_MoO_6_ and the incoming ions of SiO_3_^2−^ under a one-pot hydrothermal treatment [19]. However, the high temperature and pressure of the hydrothermal synthesis method are unsuitable for large-scale industrial production. Therefore, there is an urgent need to synthesize a Bi_2_MoO_6_ heterojunction for which high pressure is not necessary.

In the present study, we reported a type II Bi_2_MoO_6_/Bi_2_SiO_5_ heterojunction (BMOS) for the efficient degradation of RhB and TC, which was prepared via a facile in situ synthesis method. Numerous characterization techniques, including XRD, SEM, and XPS, were used to characterize the samples. The prepared sample of BMOS dramatically displayed the ascendant photodegradation performance of RhB and TC. The enhanced photocatalytic efficiency can be attributed to the BMOS with a wide photo-response range, efficient separation, and transfer of the photogenerated carriers on the heterojunction interfaces [20]. This work provides an efficient solution for improving degradation performance and broadening the application of Bi-based materials.

## 2. Materials and Methods

### 2.1. Chemicals and Reagents

The details of all reagents are shown in Supplementary Materials Text S1.

### 2.2. Preparation of Catalysts

**Figure 1:**
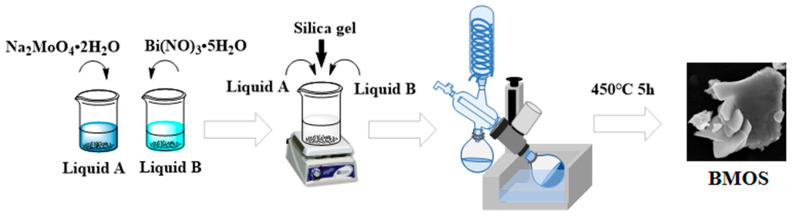
The schematic illustration of the preparation process of BMOS-x.

BMSO was prepared via an in situ synthesis method (Figure 1). As per usual, 1 mmol Na_2_MoO_4_·2H_2_O was dissolved in 15 mL HNO_3_ (1 mol/L) aqueous solution, named A. A certain amount of Bi(NO_3_)_3_·5H_2_O has dissolved in 10 mL HNO_3_ (1 mol/L) water solution, named B. A and B were sonicated for 0.5 h, then stirred continuously for 2 h. Then, A and B were mixed, and silica gel (LUDOX HS-40) was added to the solution. The solution was dried with a rotary evaporator at 90 °C to collect the precipitation. Then, the above precipitation was transferred to the Muffle furnace and calcined at 450 °C for 5 h [12]. After waiting for the resulting product to cool naturally, it was washed with ethanol and ultrapure water and centrifuged. Lastly, BMOS was obtained after drying at 60 °C for 12 h. The mixtures that were collected were called BMOS-x, where x denotes the molar ratio of Mo: Si (1:1, 2:1, 3:1, and 4:1). Bi_2_MoO_6_ (BMO) was prepared via the above method without adding a silicon source. The synthesis methods of Bi_2_SiO_5_ (BSO) are presented in Supplementary Materials Text S2.

### 2.3. Characterization

Detailed information is provided in Supplementary Materials Text S3.

### 2.4. Photocatalytic Degradation Experiment

The photodegradation properties of the compounds were investigated via RhB and TC degradation experiments. Specific details of the photocatalytic degradation experiment and total organic carbon (TOC) analysis are presented in Supplementary Materials Text S4.

### 2.5. Photoelectrochemical Measurements

Detailed information is provided in Supplementary Materials Text S5.

## 3. Results and Discussion

### 3.1. Material Characterization

**Figure 2:**
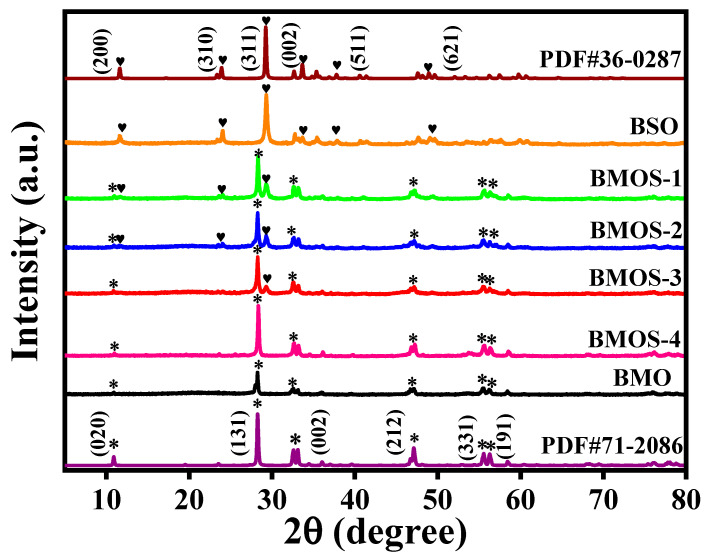
XRD patterns of BMO, BSO, and BMOS-x. “✱” and “❤” represent the different crystal faces of BMO (JCPDS: 71-2086) and BSO (JCPDS: 36-0287), respectively.

The crystalline structure of the samples was analyzed via X-ray diffraction (XRD) [21]. As shown in Figure 2, the characteristic peaks of pristine BMO are located at 2θ = 10.89°, 28.25°, 33.50°, 47.07°, 55.56°, and 56.20°, which are indexed to the (020), (131), (200), (212), (133), and (191) planes (JCPDS: 71-2086). BSO shows distinctive peaks at 2θ = 11.62°, 23.90°, 29.23°, 33.64°, 37.77°, and 52.08°, respectively, which are indexed to (200), (310), (311), (002), (511), and (621) planes of the standard card (JCPDS: 36-0287) [22]. No other peaks were observed in the BMO and BSO, indicating that they were successfully synthesized. In addition, the characteristic peaks of BSO became stronger with the dosage of Si, indicating that the BMOS-x heterojunctions were successfully prepared.

**Figure 3:**
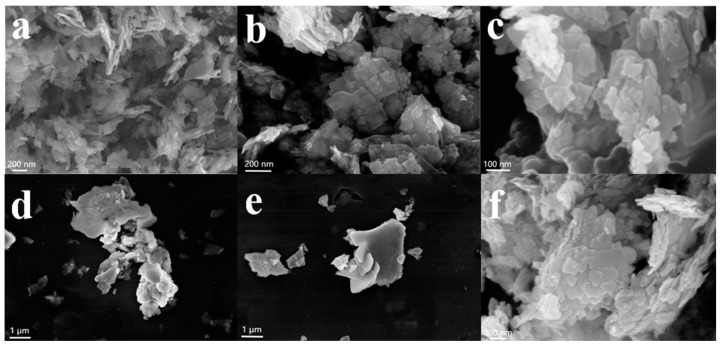
SEM micrographs of (**a**) BSO, (**b**) BMO, (**c**) BMOS-1, (**d**) BMOS-2, (**e**) BMOS-3, and (**f**) BMOS-4.

The shape and microstructure of the specimens were tested via scanning electron microscopy (SEM) [21]. As shown in Figure 3, the samples have a sheet-like morphology with some small irregular particles. Due to the surface of the lamellar structure with a certain surface energy, some particles appeared to agglomerate. The average particle diameter of BMOS-3 can be calculated to be about 83 nm (Figure S1) [23].

The surface area is an essential factor affecting the adsorption and catalytic performance of photocatalysts. The growth of BSO on the surface of BMO increased the surface areas of BMSO heterojunctions compared to pure BMO. BMOS-x showed type-IV isotherms with hysteresis lines, indicating the presence of mesoporous structures in the BMOS heterostructures (Figure S2). The mesoporous structure and high specific surface areas of BMOS heterostructures might provide a large number of active sites to enhance their photocatalytic activity.

**Figure 4:**
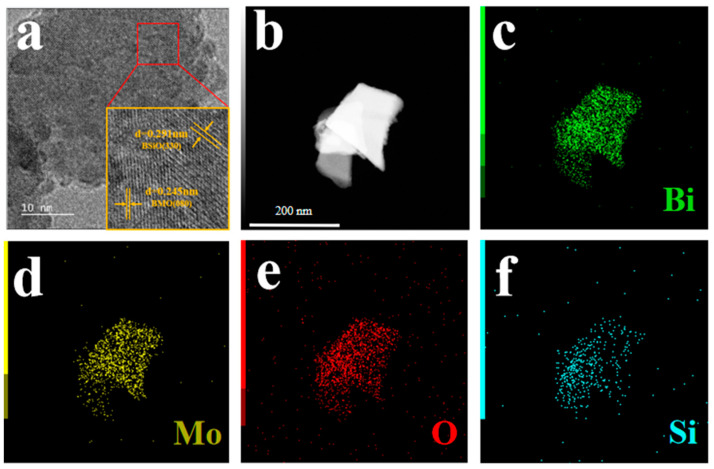
HRTEM images (**a**) and EDX analysis (**b**–**f**) of sample BMOS-3.

Energy dispersive spectroscopy (EDS) and transmission electron microscopy (TEM) images are shown in Figure 4. The BMO nanosheets were combined with BSO, reflecting that compact interfaces were formed between BMO and BSO (Figure 4a). Furthermore, the lattice of the samples that was spaced by 0.291 nm and 0.245 nm corresponds to the (330) of BMO and (080) crystal planes of BSO, respectively [24]. The distributions of the elements in the BMOS-3 were investigated via EDS in Figure 4b–f. It can be seen that Bi, Mo, O, and Si were uniformly distributed on the interface of BMOS-3. This result indicated the successful construction of BMOS-x heterojunctions.

**Figure 5:**
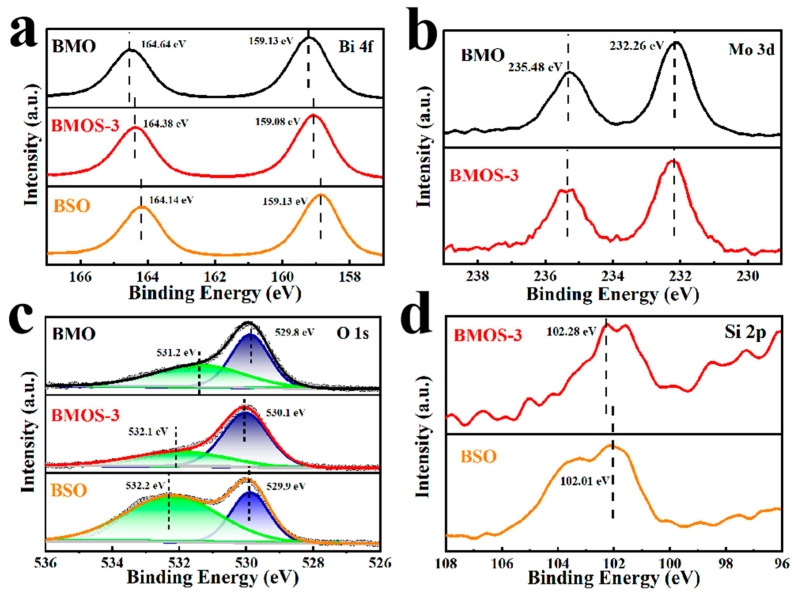
XPS spectra of BMO, BSO, and BMOS-3: high resolution of (**a**) Bi 4*f*, (**b**) Mo 3*d*, (**c**) O 1*s*, and (**d**) Si 2*p*.

The valence and electronic states of surface elements were analyzed via X-ray photoelectron spectroscopy (XPS). The presence of Bi, Si, O, Mo, and C in the samples was indicated by full-scan XPS spectra (Figure S3). XPS spectra of Bi 4*f* (Figure 5a), Mo 3*d* (Figure 5b), O 1*s* (Figure 5c), and Si 2*p* (Figure 5d) to elucidate the oxidation states of Bi, Mo, O, and Si, respectively. For the sample BMOS-3, the XPS signals of Bi 4*f* are found at the binding energies at 159.08 (Bi 4*f*_7/2_) and 164.38 eV (Bi 4*f*_7/2_), reflecting that Bi in BMOS-3 is presented as Bi^3+^ in Bi-O [25]. The binding energies of 232.26 eV (Mo 3*d*_5/2_) and 235.48 eV (Mo 3*d*_3/2_) are detected for Mo 3*d*, indicating that the state of Mo is Mo^6+^ [11]. The binding energy of 102.01 eV corresponding to Si 2*p* is detected, indicating that Si exists in the material as Si^4+^ [26]. Additionally, the two characteristic peaks at 530.1 and 532.1 eV correspond to the XPS signals of O 1*s*, which are contributed by the lattice oxygen and hydroxyl oxygen of BMOS-3, respectively. The hydroxyl oxygen is derived from hydroxyl groups on the surface or water adsorbed on the surface, while the lattice oxygen is composed of Bi-O-Bi. The slight shift in the O 1*s* binding energies among the samples could be attributed to the presence of different chemical environments of O species [27]. Compared with BSO, the peak of O 1*s* in BMOS-3 was shifted to higher binding energies, indicating that the chemical environment of Si has changed. This is caused by the close interaction between BMO and BSO. The change in the chemical environment of the elements in the XPS spectra indicates that the BMOS-x heterojunctions were prepared successfully.

### 3.2. Photocatalytic Activity

**Figure 6:**
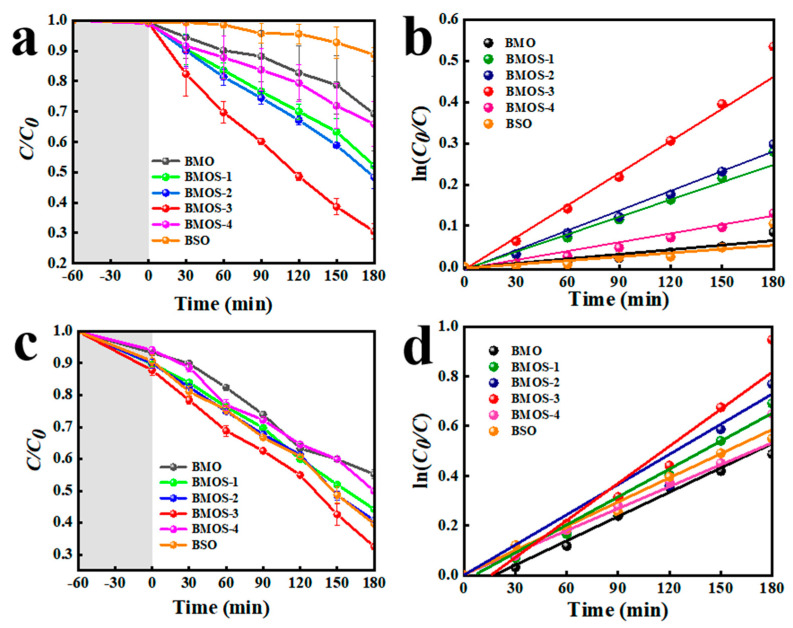
Photocatalytic degradation and pseudo-first-order rate constants of (**a**,**b**) RhB and (**c**,**d**) TC by various photocatalysts under visible light. Reaction conditions: RhB = 0.02 mmol/L, TC = 0.04 mmol/L, catalyst = 0.6 g/L.

We synthesized several BMOS-x heterojunction photocatalysts and also investigated their activities in RhB (Figure 6a,b) and TC (Figure 6c,d) photodegradation by using a 500 W Xe lamp as the optical source [28]. Compared with BMO and BSO, the degradation capacity of RhB by BMOS-3 was 7.5 times higher than that of BSO and 3.75 times higher than that of BMO. The degradation capacity of TC by BMOS-3 was 1.58 times that of BMO and 1.42 times that of BSO. As illustrated, the degree of photodegradation relies on the BSO content, and we identified BMOS-3 as the most effective composite (75% RhB and 62% TC in 180 min). To further explore its photocatalytic activity, the degradation kinetics of the BMOS-x catalysts were modeled by a pseudo-first-order model (Figure 6b,d). Figure S4 gives the photocatalytic degradation rates of RhB (TC) under visible light on the BMO, BSO, and BMOS-x. BMOS-3 showed the optimal photocatalytic degradation rate. In the BMOS-3/light system, 45.3% (RhB) and 34.4% (TC) of the total organic carbon (TOC) can be eliminated. In addition, the BMOS-3 heterojunction also showed better photocatalytic activity for the photodegradation of RhB compared with some previous reports (Table S1).

**Figure 7:**
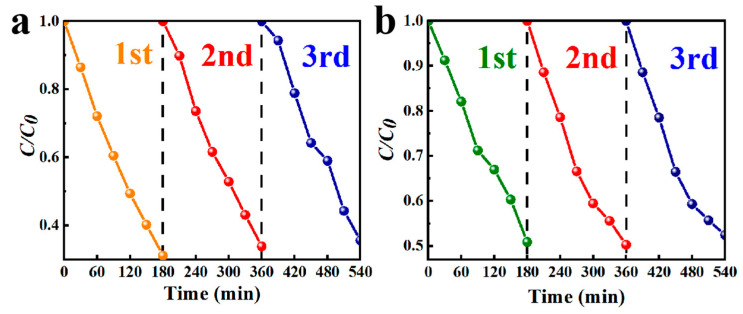
Cyclic operation of photocatalytic degradation of (**a**) RhB and (**b**) TC in the existence of BMOS-3.

The stability of the material is an essential element in determining its future commercialization and industrialization. The stability of BMOS-3 was revealed via cycling experiments. In Figure 7a,b, BMOS-3 still maintained a stable degradation capacity of 65% (RhB) and 49% (TC) after three stability experiments, respectively.

### 3.3. Possible Photocatalytic Mechanism

**Figure 8:**
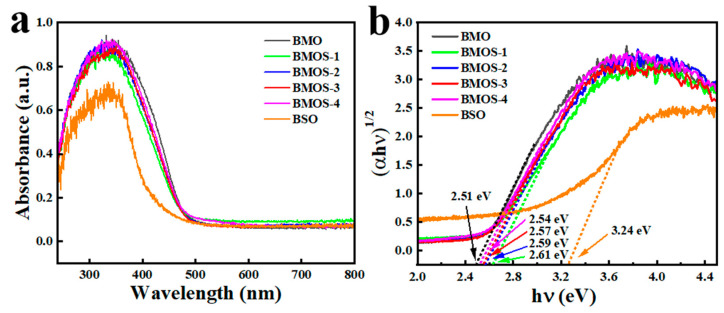
(**a**) UV–VIS DRS spectra and (**b**) Tauc plots of BMO, BSO, and BMOS-X.

As shown in Figure 8a,b, the optical absorption properties and band gaps of the as-prepared heterojunctions were investigated via UV-VIS spectroscopy. As shown in Figure 8a, with the increase in the BSO component content, the absorption edges that occurred slightly blue-shifted, and the absorbance gradually became stronger, suggesting that more visible light energy can be absorbed due to the successful formation of heterojunctions. The band gap energies (Eg) were computed with the Tauc plots [9]. In Figure 8b, the band gap energies of BMO and BSO were 2.51 and 3.24 eV, respectively.

**Figure 9:**
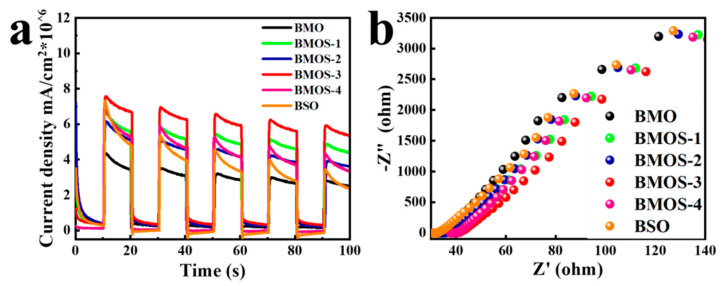
(**a**) Transient photocurrent responses and (**b**) EIS plots of BMO, BSO, and BMOS-x.

The carrier separation efficiency was evaluated via photocurrent response spectroscopy and electrochemical impedance spectroscopy (EIS) analysis [29]. As shown in Figure 9a, BMOS-3 produced the highest photocurrent density with a photocurrent intensity of 8 μA/cm^2^ (which was twice that of BMO), indicating that this photocatalyst has predominant photoelectric separation and conversion efficiency.

Furthermore, the charge transfer capability of the samples was further investigated via EIS to quantify their electron transfer efficiencies [30]. As shown in Figure 9b, the arc radius of pure BMO and BSO is larger than that of the BMOS-3 photocatalyst, indicating that the internal resistance of the material can be reduced by constructing a heterojunction [31].

**Figure 10:**
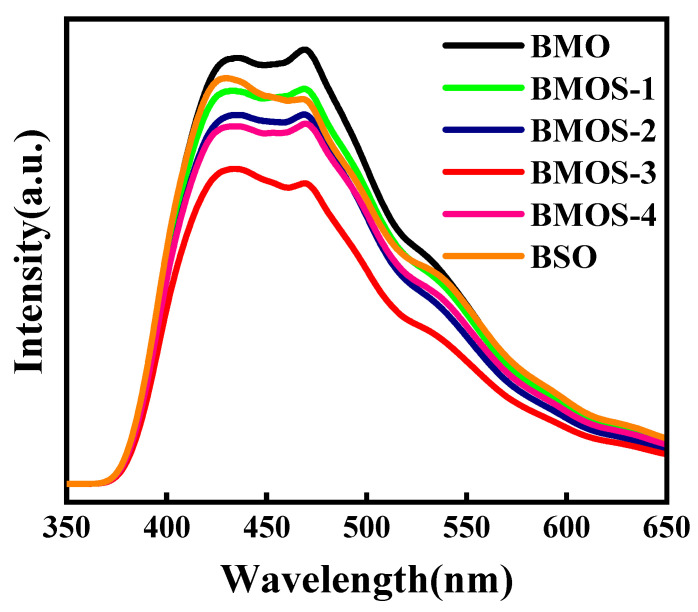
PL spectra of BMO, BSO, and BMOS-x.

The separation rate of the photogenerated electrons and holes was subsequently evaluated via PL emission intensity [32]. As shown in Figure 10, the PL emission intensity of BMOS-x was obviously lower than those of the BMO and BSO samples, indicating that the conjunction formation improved electron–hole separation efficiency.

**Figure 11:**
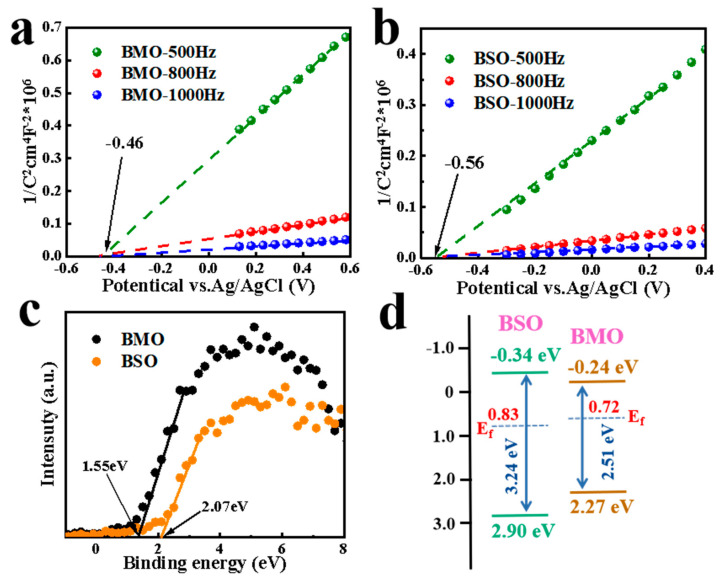
Plot of Mott−Schottky curves at different frequencies (**a**) BMO and (**b**) BSO. (**c**) VB-XPS spectrum. (**d**) Schematic of the energy band structure of BMO and BSO.

Both Mott–Schottky (Figure 11a,b) and VB-XPS tests (Figure 11c) were performed on the samples to determine the conduction, valence, and energy band structures of the samples. These samples are n-type semiconductors attributed to the positive slopes of the C^−2^ potential. Furthermore, the flat band potentials of BMO and BSO are −0.46 V and −0.56 V versus Ag/AgCl, respectively. Therefore, the E_CB_ values of BMO and BSO are correspondingly −0.24 eV and −0.34 eV versus NHE. As shown in Figure 8b, the forbidden bandwidths of BMO and BSO are 2.51 eV and 3.24 eV, respectively. Therefore, the valence band potentials of BMO and BSO are equivalent to 2.27 eV and 2.90 eV versus NHE.

The distances from the Fermi level to the valence band can be obtained from the VB-XPS spectra (Figure 11c). The Fermi levels of BMO and BSO can be further computed as 0.72 eV and 0.83 eV, respectively [33]. As shown in Figure 11d, the energy level structure of the compounds can be drawn according to the above calculated values.

**Figure 12:**
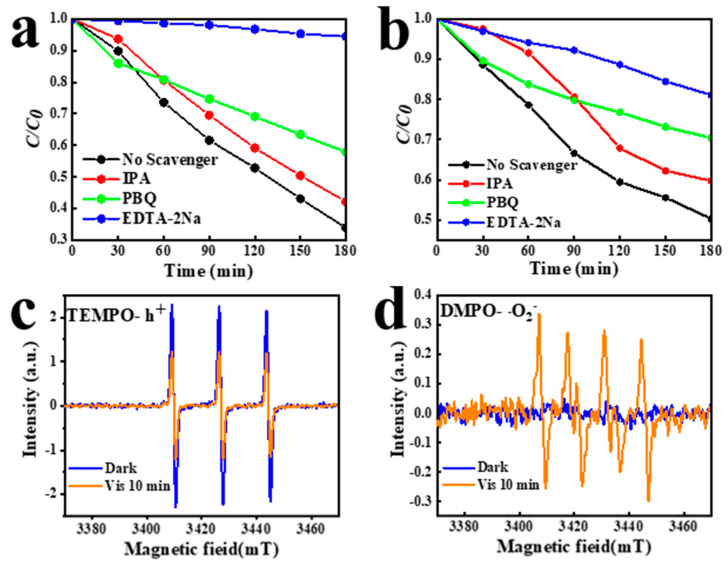
Influence of various scavengers on the photodegradation efficiency of BMOS-3 (**a**) degradation of RhB and (**b**) TC, ESR spectra; (**c**) TEMPO-$h^{+}$; and (**d**) DMPO-•$O_{2}^{-}$.

IPA, EDTA-2Na, and PBQ were used as scavengers of •OH, $h^{+}$ and •$O_{2}^{-}$, respectively, in trapping experiments to determine the active species in the photodegradation of RhB and TC [34]. As shown in Figure 12, the RhB and TC degradation rates decreased to 60% and 40%, respectively, after IPA addition. When using PBQ as sacrificial agents of •$O_{2}^{-}$, the reactivity was further hindered, and the degradation capacity was further reduced to 40% and 30%, respectively. The photodegradation efficiency of RhB and TC decreased significantly with the addition of EDTA-2Na.

ESR tests were used to further investigate the active species produced by BMOS-3 during photodegradation. TEMPO and DMPO were used as active species-trapping agents to capture $h^{+}$ and •$O_{2}^{-}$, respectively. As shown in Figure 12c, no obvious signals were observed in the dark. Under open light conditions, the clear signal of TEMPO-$h^{+}$ can be observed with the intensity ratio of 1:1:1 for the quadratic peaks. The six peak signals of DMPO-•$O_{2}^{-}$ are also clearly observed in Figure 12d [35]. Based on the tests above, $h^{+}$ plays a dominant role in the photocatalytic degradation of RhB and TC, while •$O_{2}^{-}$ plays a secondary role.

The photocatalytic reactions can be briefly described as follows:

(1) $$\mathrm{BMO}+hv\;\to \;\mathrm{BMO}\;(e^{-}{+h}^{+})$$

(2) $$\mathrm{BSO}+hv\;\to \;\mathrm{BSO}\;(e^{-}{+h}^{+})$$

(3) $$O_{2}+e^{-}\;\to {\;•O}_{2}^{-}$$

(4) $${\mathrm{RhB}+•O}_{2}^{-}{,\;h}^{+}\;\to {\;\mathrm{CO}}_{2}+H_{2}O$$

(5) $${\mathrm{TC}+•O}_{2}^{-}{,\;h}^{+}\;\to {\;\mathrm{CO}}_{2}+H_{2}O\;$$

**Figure 13:**
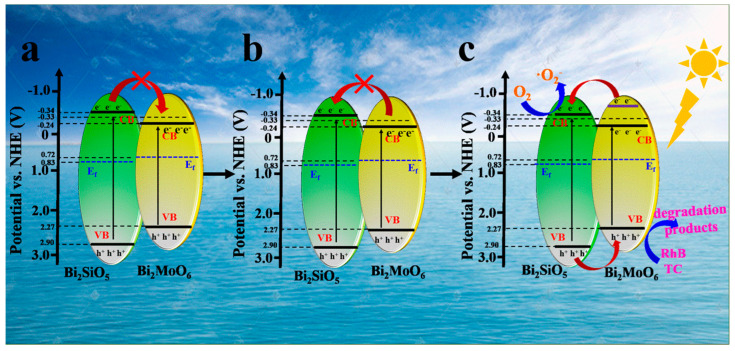
Mechanism of photocatalytic degradation of BMOS-3.

According to the above experimental results and data analysis, a possible mechanism for the photocatalytic degradation of RhB and TC by BMOS-3 was proposed. As shown in Figure 13a, it can be found that the conduction band of BSO is more negative than that of BMO. This implies that electrons may transfer from BSO to BMO. The conduction band position of BMO was observed to be −0.24 eV, while the •$O_{2}^{-}$ potential (O_2_/•$O_{2}^{-}$) was −0.33 eV. It is not sufficient to generate the •$O_{2}^{-}$ active species. Furthermore, the Fermi level of BMO is higher than that of BSO, which is contrary to the electron flow. Therefore, the inference in Figure 13a is not reasonable. As shown in Figure 13b, electron transfer from BMO to BSO is not possible because the conduction band of BSO is more negative than that of BMO. Then, we hypothesize the existence of high-energy electron orbitals in BMO, as reported previously [36]. Photogenerated electrons transferred from the high-energy electron orbitals to the conduction band of BSO. The electrons combine with O_2_ to form •$O_{2}^{-}$. Meanwhile, As shown in Figure 13c, $h^{+}$ transferred from the valence band of BSO to the valence band of BMO and participated in the oxidative degradation of RhB and TC. Thus, successfully establishing BMOS-x can prevent electron–hole complexation, promote photogenerated carrier transfer, and improve photocatalytic activity.

## 4. Conclusions

In conclusion, BMOS were prepared via a facile in situ synthesis method and showed excellent photocatalytic degradation activity of RhB and TC. The efficiency of the photocatalytic degradation of RhB by BMOS-3 reached 75% within 180 min, which is 7.5 times that of BSO and 3.75 times that of BMO. The degradation capacity of TC by BMOS-3 was 62%, which is 1.58 times that of BMO and 1.42 times that of BSO. The reasons for this result can be attributed to the fact that the loading of BSO modulates the energy band structure of the semiconductor. Moreover, the close contact between BSO and BMO increases electron transport efficiency and improves electron utilization. Finally, the successful construction of type II heterostructures prevents electron–hole complexation, promotes photogenerated carrier transfer, and enhances photocatalytic efficiency. Overall, this material would have a wide range of applications in the photocatalytic degradation of persistent pollutants [37].

## Data Availability

Not applicable.

## Supplementary Materials

The following supporting information can be downloaded at: https://www.mdpi.com/article/10.3390/ma16103631/s1. Text S1. Chemicals and Reagents; Text S2. Synthesis of BSO; Text S3. Characterization; Text S4. Photocatalytic degradation experiment; Text S5. Photoelectrochemical Measurements; Figure S1. SEM images particle size distribution of (a) BMO, (b) BSO, and (c) BMOS-3. Figure S2. N_2_ adsorption–desorption isotherms of BMO, BSO, and BMOS-x. Figure S3. XPS full spectra of BMO, BSO, and BMOS-3. Figure S4. Photocatalytic degradation rates of (a) RhB and (b) TC on BMO, BSO, and BMOS-x. Figure S5. The photograph of photocatalytic degradation experimental instrument. Table S1. Photocatalytic efficiency of RhB over various photocatalysts. Table S2. R^2^ value in Figure 6b,d. References [38,39,40] are cited in the supplementary materials.
