# Supplementary material for: In Situ Synthesis of Bi2MoO6/Bi2SiO5 Heterojunction for Efficient Degrading of Persistent Pollutants
Source: Materials (Basel). 2023 May 10;16(10):3631. doi: 10.3390/ma16103631 (PMC10223800; doi:10.3390/ma16103631)
Supplement: Supplementary file 1 [file materials-16-03631-s001.zip › materials-2332564-supplementary.pdf]

Supplementary Materials

In Situ Synthesis of Bi_2MoO_6/Bi_2SiO_5 Heterojunction for Efficient Degrading of Persistent Pollutants

Kaiwen Yuan [1], Hailong Jia [1], Daimei Chen [1,*], Yanmei Feng [1], Yu Liang [2,*], Kai Chen [3] and Derek Hao [4,*]

[1] Engineering Research Center of Ministry of Education for Geological Carbon Storage and Low Carbon Utilization of Resources, China University of Geosciences, Xueyuan Road, Haidian District, Beijing 100083, China; 2103210004@email.cugb.edu (K.Y.)

[2] School of Materials Science and Technology, Shenyang University of Chemical Technology, Shenyang 110142, China

[3] Collaborative Innovation Center of Atmospheric Environment and Equipment Technology, Jiangsu Key Laboratory of Atmospheric Environment Monitoring, Pollution Control School of Environmental Science and Engineering, Nanjing University of Information Science and Technology, Nanjing 210044, China; catqchen@163.com

[4] School of Science, STEM College, RMIT University, Melbourne, VIC 3000, Australia

* Correspondence: chendaimei@cugb.edu.cn (D.C.); liangyuaadd@126.com (Y.L.); haoqiangderek@gmail.com (D.H.)

This document contains the following:

1. Text

2. Figures

3. Tables

4. References

Text S1. Chemicals and Reagents

Sodium molybdate dihydrate (Na_2MoO_4, 99.7%), bismuth nitrate pentahydrate ($Bi(NO_3)_3·5H_2O$, 99.7%), nitric acid (HNO_3, 99.7%), Rhodamine B (RhB, $C_{28}H_{31}ClN_2O_3$, 99.7%), and ethylenediaminetetraacetic acid disodium salt dihydrate (EDTA-2Na, 99.0%) were obtained from Sinopharm Chemical Reagent Co., Ltd. (Shanghai, China). Ethylene glycol (EG, $C_2H_6O_2$, 99.5%) and isopropanol (IPA, C_3H_8O, 99.7%) were purchased from Macklin Chemistry Co., Ltd. (Shanghai, China). Silica gel (LUDOX HS-40, 40wt.%) was obtained from Shanghai Shuanglun Industrial Co., Ltd. (Shanghai, China). Sodium metasilicate nonahydrate ($Na_2SiO_3·9H_2O$, 99.7%) was obtained from Tianjin Guangfu Technology Development Co., Ltd. (Tianjin, China). Para-benzoquinone (p-BQ, $C_6H_4O_2$, 99.0%) was purchased from Aladdin Reagent Co., Ltd. (Shanghai, China). Ethanol (EtOH, C_2H_6O, 99.7%) was obtained from the Beijing Chemical Industry Group Co., Ltd. (Beijing, China). Tetracycline hydrochloride (TC, $C_{22}H_{25}ClN_2O_8$, 99.0%) was obtained from Beijing Solarbio Science & Technology Co., Ltd. (Beijing, China). All reagents were used without further purification.

Text S2. Synthesis of BSO

BSO was prepared via the melting calcination method. A total of 25 mmol $Bi(NO)_3·5H_2O$ was dissolved in 35 mL ethylene glycol and sonicated for 0.5 h (liquid A). In total, 2 mmol $Na_2SiO_3·9H_2O$ was dissolved in 5 mL distilled water and sonicated for 0.5 h (liquid B). Liquid A was then blended dropwise into liquid B under continuous stirring. The mixture was continuously stirred for 1 h, then transferred to a 50 mL Teflon-lined stainless-steel autoclave. The solution was synthesized by maintaining the autoclave at 180 °C for 8 h. After the solution cooled to ambient temperature, BSO precursors were collected by washing with deionized water and ethanol and then dried at 100 °C for 4 h. The dried precursor was ground and transferred to a crucible, then calcined in a muffle furnace at 500°C for 12 hours. After natural cooling, BSO was collected by washing with EtOH and water and then dried at 60 °C overnight.

Text S3. Characterization

The crystalline properties of all as-synthesized samples were determined via X-ray diffraction (XRD, Bruker D8 Advance) with a Cu Kα radiation source (λ = 0.15406 nm) in the 2θ range of 5°-80° at a rate of $2°·min^{-1}$. Scanning electron microscopy (SEM,

Field Emission Scanning Electron Microscope SU8010) was used to examine the morphologies of samples. The morphology and crystal lattice parameters were ascertained via transmission electron microscopy (TEM, JEM-2100F). The elemental composition of the synthesized samples was obtained via X-ray photoelectron spectroscopy (XPS, Thermo Scientific K-Alpha). The photoluminescence (PL) spectra of all photocatalysts were investigated by using a steady-state/transient fluorescence spectrometer (F-4700, Hitachi, Japan). The optical properties and band gap energies were determined via UV-VIS diffuse reflectance spectra (DRS, Shimadzu UV-3900). Electron spin resonance (ESR) signals were obtained with an EMXnano spectrometer (Bruker, Germany) by using 5,5-dimethyl-1-pyrroline-N-oxide (DMPO) and 2,2,6,6-Tetramethyl-1-piperidinyloxy (TEMPO) as the trapping agent.

Text S4. Photocatalytic degradation experiment

The photodegradation properties of the compounds were investigated via RhB and TC degradation experiments (Figure S5 right image). Photocatalytic degradation experiments were conducted by using the Phchem III series photochemical reactor (Beijing NBET Technology Co., Ltd., China). A 0.04 mmol/L TC solution (50 mL) or 0.02 mmol/L RhB solution (50 mL) was placed in a quartz test tube, and the photocatalyst (30 mg) was added. Prior to illumination, the solution and photocatalyst were thoroughly stirred to achieve adsorption–desorption equilibrium. The photocatalytic performance tests were carried out under the illumination of a 500 W xenon lamp (XE-JY500, Beijing NBET Technology Co., Ltd., China) for 3 h. The xenon lamp was used without the addition of optical filters to simulate solar radiation. During illumination, an aliquot (4 mL) of the sample was collected every 0.5 h. The photocatalytic reaction efficiency was obtained via Eq. (S1).

$$\eta = C/C_0 \quad \text{(S1)}$$

where η is photocatalytic performance efficiency, C_0 is the initial absorbance of the pollutant, and C is the absorbance of pollutants at different times, respectively.

A pseudo-first-order kinetic model was calculated via Eq. (S2) to make further comparisons with the photocatalytic efficiencies.

$$\ln(C_0/C) = kt \quad \text{(S2)}$$

where C_0 and C are the initial concentration and the remaining concentration of RhB or TC at each time point, respectively. K is the kinetics rate constant, and t is the reaction time.

In order to reduce the error caused by the volume change, we expanded the volume of the pollutant and the quality of the catalyst by a factor of ten during the photocatalytic degradation experiment (Figure S5 left image). A 0.04 mmol/L TC solution (500 mL) or 0.02 mmol/L RhB solution (500 mL) was placed in a quartz test tube, and the photocatalyst (300 mg) was added. Prior to illumination, the solution and photocatalyst were thoroughly stirred to achieve adsorption–desorption equilibrium. The photocatalytic performance tests were carried out under the illumination of a 500 W xenon lamp (Solar-500, Beijing NBET Technology Co., Ltd., China) for 3 h. A xenon lamp without the addition of optical filters was used to simulate solar radiation. During illumination, an aliquot (4 mL) of the sample was collected every 0.5 h. Because our degradation system was homogeneous, it did not have much influence on the measurement results.

Total organic carbon (TOC) analysis was carried out by adding 30 mg BMOS-3 (BMO and BSO) into 50 mL RhB (TC) aqueous solution (80 mg/L). After dark adsorption for 60 min and light exposure for 180 min, the degraded products were collected and filtered by the membrane pore size of 0.45 μm to remove the photocatalyst and were then detected by a Shimadzu TOC-L total organic carbon analyzer.

Text S5. Photoelectrochemical Measurements

Photoelectrochemical measurements were measured on an electrochemical system (CHI 660E, Shanghai Chenhua Instrument Co., Ltd., China). A typical three-electrode was immersed in a 0.1 mol·L^{-1} Na_2SO_4 electrolyte solution. Fluorine-doped tin oxide (FTO)-conductive glass was employed as the working electrode; Pt and Ag/AgCl electrodes were employed as the counter and reference electrodes, respectively. The working electrode was prepared as follows: 10 mg of catalysts was first mixed with 1 mL of DI water to produce a slurry. Afterward, the slurry was pipetted onto a piece of FTO glass and then dried at 60 °C. The photocurrent response was recorded by using a 500 W xenon lamp (Solar-500, Beijing NBET Technology Co., Ltd., China) equipped with a 420 nm cutoff filter. The electrochemical impedance spectroscopy (EIS) was performed at the open circuit potential with a frequency range from 1 Hz to 10000 Hz under irradiation.

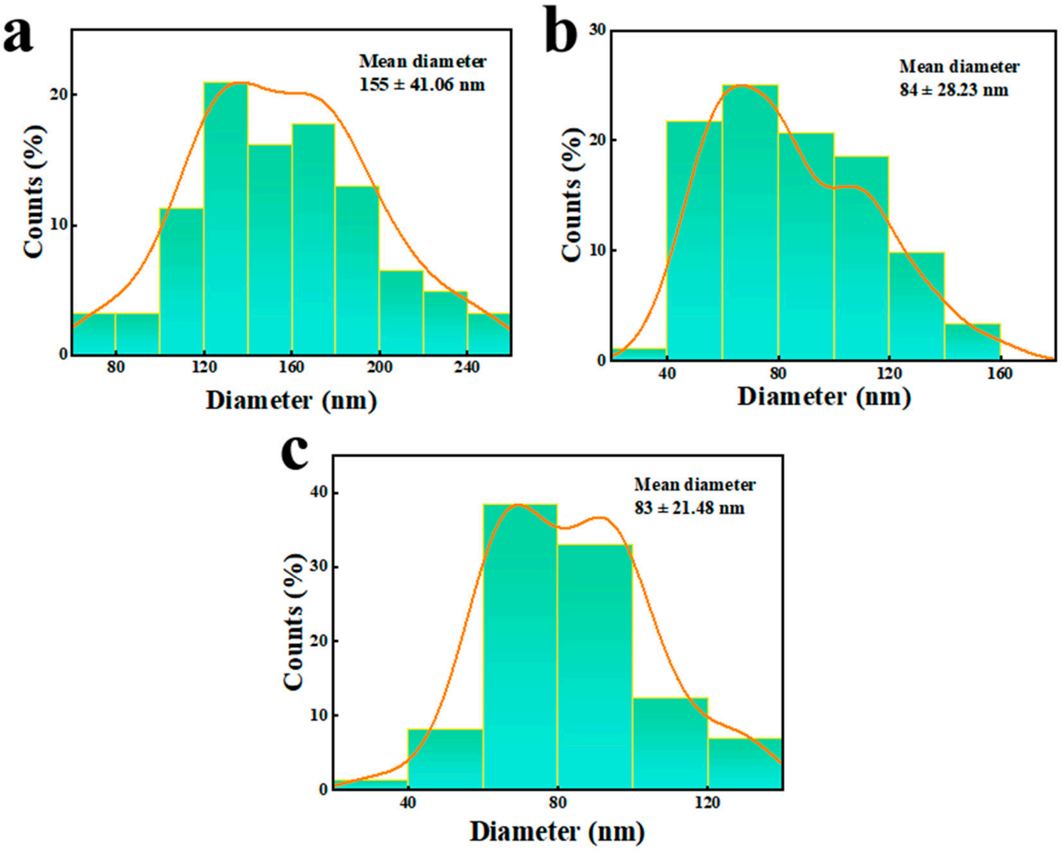

Figure S1. SEM image particle size distribution of (a) BMO, (b) BSO, and (c) BMOS-3.

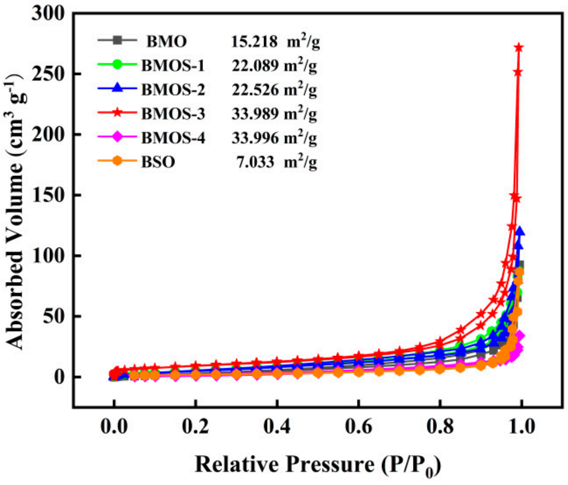

Figure S2. N_2 adsorption–desorption isotherms of BMO, BSO, and BMOS-x.

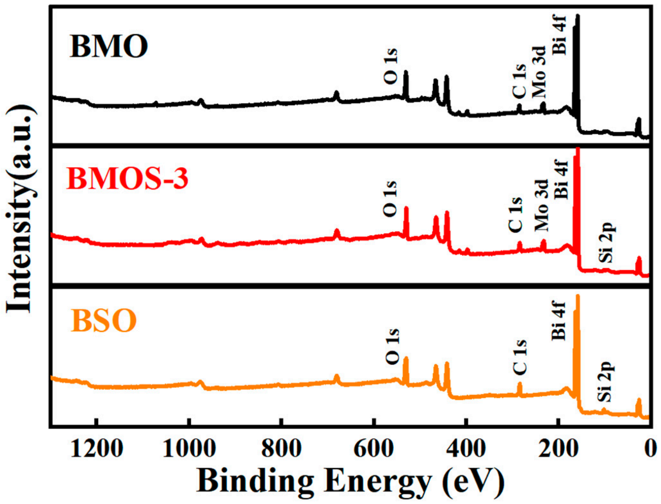

Figure S3. XPS full spectra of BMO, BSO, and BMOS-3.

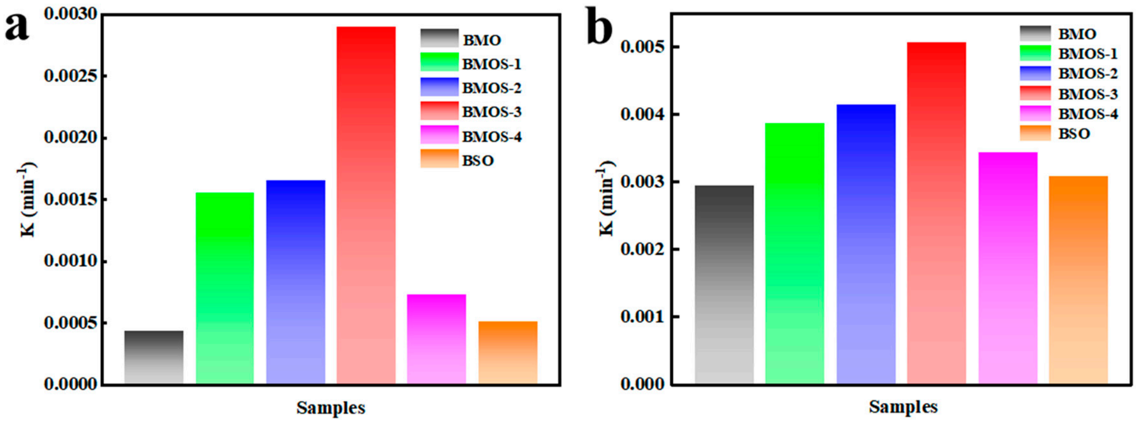

Figure S4. Photocatalytic degradation rates of (a) RhB and (b) TC on BMO, BSO, and BMOS-x.

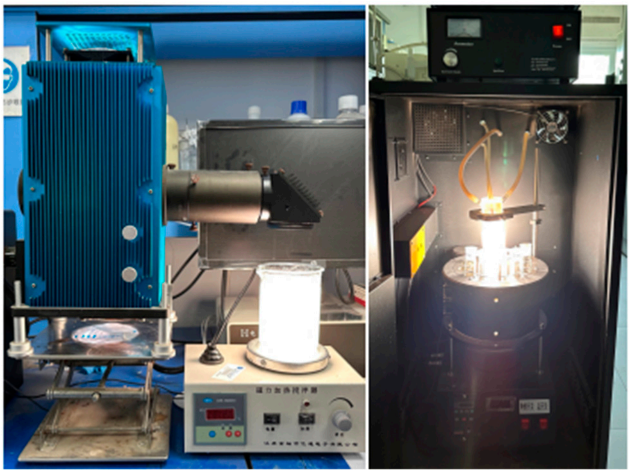

Figure S5. The photograph of photocatalytic degradation experimental instrument.

Table S1. Photocatalytic efficiency of RhB over various photocatalysts.

Photocatalyst	C_{RhB}	Photocatalytic efficiency	Ref.
BMOS-3	0.02 mM	75% 180 min	This work
$BiIO_4/Bi_2MoO_6$	0.01 mM	55% 300 min	[1]
ZnO–SnO	0.01 mM	49% 300 min	[2]
$BiFeO_3$/BiOCl	0.02 mM	68% 75 min	[3]

Table S2. R^2 value in Figure 6b and 6d.

Pollutant	RhB					
Samples	BMO	BMOS-1	BMOS-2	BMOS-3	BMOS-4	BSO
R^2	0.90936	0.98954	0.99027	0.98642	0.97341	0.97829

Pollutant	TC					
Samples	BMO	BMOS-1	BMOS-2	BMOS-3	BMOS-4	BSO
R^2	0.98364	0.98164	0.96051	0.95414	0.98252	0.99386

References

[1] Huang, H.; Liu, L.; Zhang, Y.; Tian, N. One pot hydrothermal synthesis of a novel $BiIO_4/Bi_2MoO_6$ heterojunction photocatalyst with enhanced visible-light-driven photocatalytic activity for rhodamine B degradation and photocurrent generation. *J. Alloys Compd.* **2015**, *619*, 807-811.

[2] Pascariu, P.; Airinei, A.; Olaru, N.; Olaru, L.; Nica, V. Photocatalytic degradation of Rhodamine B dye using $ZnO–SnO_2$ electrospun ceramic nanofibers. *Ceram. Int.* **2016**, *42*, 6775-6781.

[3] Shang, J.; Chen, H.; Chen, T.; Wang, X.; Feng, G.; Zhu, M.; Yang, Y.; Jia, X. Photocatalytic degradation of rhodamine B and phenol over $BiFeO_3/BiOCl$ nanocomposite. *Appl. Phys. A* **2019**, *125*, 1-7.
